# Exploring Views of Healthcare Professionals, Researchers, and People Living with and beyond Colorectal Cancer on a Healthy-Eating and Active Lifestyle Resource

**DOI:** 10.3390/nu11102482

**Published:** 2019-10-16

**Authors:** Jana Sremanakova, Debra Jones, Richard Cooke, Sorrel Burden

**Affiliations:** 1 School of Health Sciences, University of Manchester, Oxford Road, Manchester M13 9PL, UK; debra.jones@manchester.ac.uk (D.J.); sorrel.burden@manchester.ac.uk (S.B.); 2 Department of Psychology, University of Liverpool, Bedford Street South, Liverpool L69 7ZA, UK; R.Cooke4@liverpool.ac.uk

**Keywords:** colorectal, cancer, diet, physical activity, behavior change, survivorship

## Abstract

Background: People after bowel cancer are at high risk of cancer recurrences and co-morbidities, and therefore strategies are needed to reduce these risks. One promising strategy targets modifiable lifestyle factors including diet and physical activity. However, effective, evidence-based resources in adopting new lifestyle habits are currently lacking. Methods: The Healthy-Eating and Active Lifestyle After Bowel Cancer (HEAL ABC) resource was developed incorporating behavior change theory and World Cancer Research Fund and American Institute of Cancer Research guidelines. Focus groups and telephone interviews were conducted with professionals and survivors (age ≥18 years) to obtain feedback on the resource layout, structure, and content. Recorded data were transcribed verbatim and analyzed using framework analysis. Results: Thirty participants evaluated the resource—19 cancer survivors and 11 professionals. Survivors’ mean age was 62 years (SD 11.5), 11 (58%) were females and 8 (42%) were male. Professionals were all females and mean age was 40 years (SD 6.06). Both survivors and professionals evaluated the resource as useful and provided suggestions for improvements. Conclusions: HEAL ABC is an evidence-based resource designed to aid cancer survivors in translating their motivation into action. It was valued positively by both survivors and healthcare professionals and viewed as filling a gap in post-treatment advice.

## 1. Introduction

Bowel or colorectal cancer (CRC) is the third most commonly diagnosed cancer worldwide [1] and in the UK [2]. Due to advances in early cancer detection and treatment, the number of people surviving cancer has doubled in the last 40 years [3,4]. In the UK, this number is predicted to rise, and it is estimated that by 2024 nearly a quarter of people aged 65 years and over will be a cancer survivor [4].

People who survive cancer are at high risk of recurrence and co-morbidities [5,6], which consequently places increased demands and burden on healthcare providers and resources [7]. Mounting evidence indicates that addressing modifiable lifestyle factors such as diet and exercise can reduce the risk of diseases and improve cancer survival [8,9] meaning, tertiary cancer prevention using lifestyle interventions is becoming increasingly important.

Evidence shows that people who have received cancer treatment are more motivated to improve their lifestyle, creating an ideal opportunity to encourage behavior change [10]. Also, a recent report by the National Cancer Institute and James Lind Alliance UK [11] has identified that more research is required. This included delivering long-term cancer care, self-management and specific lifestyle changes (e.g., diet, exercise and stress reduction) to help people recover from treatment, restore health, and improve quality of life. Self-management and lifestyle modifications were named in the top research priorities by people who survived cancer.

Cancer survivors are willing to change although require support with initiation and maintenance of healthy choices [12,13]. Thus, there is an unmet need to identify the most effective tool to help survivors improve their lifestyle. It has already been recognized that this approach may involve epidemiological research to determine the best lifestyle recommendations for cancer prevention and psychological investigations on implementing behavioral change techniques, in conjunction with engagement from survivors and professionals to enable tool development [14,15]. Research indicates that self-management support using an evidence-based resource guiding survivors to improve lifestyle habits is a potential investment for healthcare funds [16] and represents a promising strategy to assist people in their actions and goal setting, to aid new habit formation [17].

However, there is a paucity of evidence for sustainable lifestyle interventions and effective intervention resources. The resources provided to survivors in most published trials are not tested for practicality, acceptability, and are not tailored to meet the needs of people after cancer [18], or specifically after CRC [19]. Most studies have focused on physiological changes but lack justification for selected modes of delivery and structure of the intervention resources and often fail to fully involve participants in their development [20]. These studies also lack details on the behavioral theories implemented, if used at all, and how these theories can be used to explain the changes observed [20]. A substantial amount of information on evidence-based resources and behavior change theory for cancer survivors comes from studies in women after breast cancer [20,21,22], but there are only a few studies on survivors of CRC. However, people after CRC indicated in semi-structured interviews that they wish to change their lifestyle [23] and had a preferred option for receiving information as written materials with support provided via telephone [24].

Hence, we have developed a healthy-eating and active lifestyle resource that includes information and techniques to promote behavior change based on theory. In this project, we aimed to evaluate the developed resource by involving survivors of CRC and professionals using focus groups and telephone interviews.

## 2. Materials and Methods

This was a qualitative study designed to evaluate the resource for people who have survived CRC.

**Figure 1:**
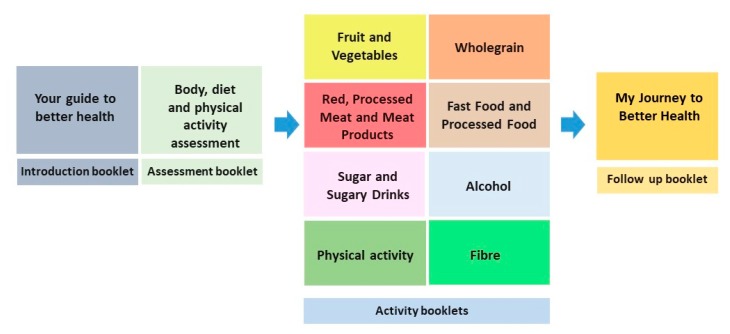
A structure of the Healthy-Eating and Active Lifestyle After Bowel Cancer (HEAL ABC) resource.

**Figure 2:**
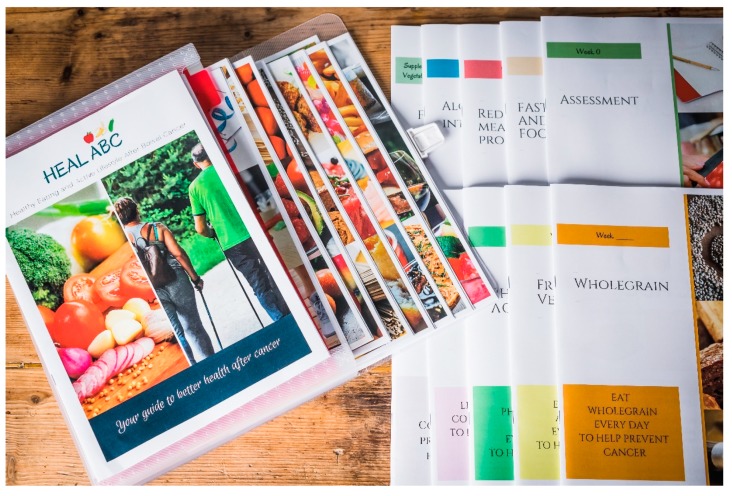
Healthy-Eating and Active Lifestyle After Bowel Cancer (HEAL ABC) resource.

The Healthy-Eating and Active Lifestyle After Bowel Cancer (HEAL ABC) is a nutrition and active lifestyle resource supporting people after CRC. The resource consists of number of workbooks (See Figure 1 and Figure 2) and provides dietary and physical activity guides to enable users to form new diet and activity habits based on the World Cancer Research Fund and American Institute Cancer Research recommendations (WCRF/AICR) [25] combined with strategies to integrate physical activity into everyday life [26]. The Health Action Process Approach (HAPA) [27] psychology model was implemented in the resource. The HAPA recognizes that adopting and maintaining a new behavior is a long-term process, which must be supported by detailed planning and confidence (i.e., self-efficacy) in one’s ability to successfully integrate the new behavior into daily life [28].

### 2.1. Population and Recruitment

#### 2.1.1. Survivors of Colorectal Cancer

We invited adults (≥18 years) who experienced CRC to participate in our research. The opportunity to participate was advertised on local and national websites, through CRC support groups and via leaflets that were distributed in outpatient clinics.

CRC survivors could express interest by contacting a researcher by email, post or by telephone. Potential participants were provided with a participant information sheet and upon agreement to participate, received the HEAL ABC resource. We asked participants to read the resource prior to the focus group and provided them with suggested themes to be discussed. For those who could not attend a focus group, we organized one to one telephone interviews. In total, 32 people expressed an interest, 15 participants attended a focus groups and four participants were interviewed over the telephone.

#### 2.1.2. Professionals

Professionals working in healthcare services or involved in research related to CRC were recruited from across England and invited to participate by email. After expressing an interest, they were sent an information sheet and if they agreed to participate, received the HEAL ABC resource prior to a focus group. In total, 16 professionals were interested in participating, 5 professionals attended a focus group and 6 were interviewed by telephone.

#### 2.1.3. Consent

Participants gave written consent before the focus group and those contacted by telephone sent written consent prior to the interview.

#### 2.1.4. Focus Groups

Focus groups were led by two researchers (JS and DJ). During the focus group participants completed anonymous demographic and lifestyle questionnaires (Appendix A and Appendix B) designed by our research team and discussed the workbook with researchers. Discussion was based on a topic guide and included feedback on structure, layout, content, thoughts on relevance to participants, potential usability and applicability in everyday lives (Appendix C). Focus groups were recorded with an encrypted voice recorder and lasted for two hours.

#### 2.1.5. Telephone Interviews

Participants who could not attend the focus group, were offered an opportunity to take part in a telephone interview. This discussion followed the same structure as the focus groups and lasted approximately one hour. Notes were taken throughout the interviews by the researcher.

Ethical approval was sought from the University Ethics Committee (UREC, Ref: 2019-6043-9696). The study was supported by funding from N8AgriFood organization.

### 2.2. Data Analysis

Descriptive statistics were performed to define participant demographics. Data from the voice recordings were transcribed verbatim and analyzed using the framework analysis. The Framework Method was used to form the qualitative content analysis, which follows a five-step process [29]. We used a familiarization to become familiar with all transcripts and identified a thematic framework by reading and rereading the transcripts. The indexing/coding was used to code all the transcripts based on the agreed thematic framework and thematic charts were produced from this coding and indexing. Coding and thematic charts were discussed and agreed by JS and DJ. The analysis presented is based on team consensus of the interpretation of the transcripts. Quotations have been used to illustrate the themes and pseudonyms for names are used to keep anonymity of participants. The age of the participants is also included in years to add some context to the quotations.

## 3. Results

We recruited 30 participants, 19 survivors and 11 professionals to the study. Three focus groups (20 participants) and 10 telephone interviews (10 participants) were performed over a two months period between June–July 2019. Concurrent data analysis was undertaken and data saturation was deemed to have occurred when no new themes were occurring in the data. We ran two focus groups with survivors (15 participants) and 1 focus group (5 participants) with healthcare professionals and researchers. Four survivors and six professionals were interviewed by telephone.

**Figure 3:**
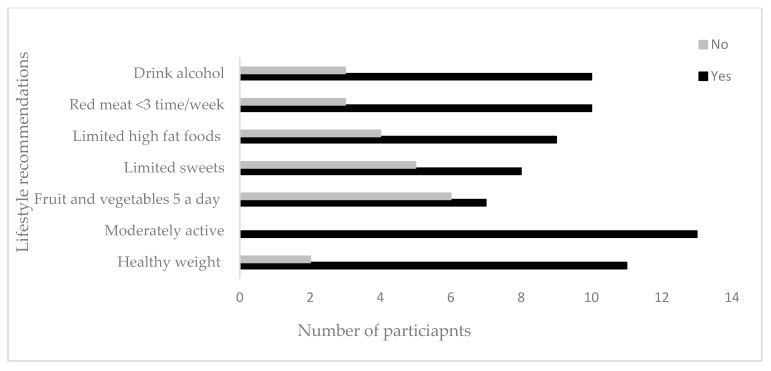
Participants’ lifestyle behaviors based on self-reported questionnaire. (*n* = 13).

The mean age of the survivors was 62 (SD 11.5) years and ranged from 35 to 74 years. Participants were from Great Manchester, Wales, and London. All the survivors where white British, 11(58%) were females and 8 (42%) were males. Thirteen participants provided information on their marital status, education, surgery (Table 1) and current lifestyle (Figure 3). The mean age of the professionals was 40 (SD 6.06) years. Professionals were all females and included dietitians, oncology nurses, occupational therapist, physiotherapists, and researchers in nutrition and psychology with mean 12 (SD 7.03) years of working experience and professionals were employed in Manchester and London.

### 3.1. General Feedback

Both professionals and CRC survivors expressed enthusiasm and positivity towards the HEAL ABC resource and its development. They appreciated the idea of helping people to improve their lifestyle: “I like the personal choice, things about self-determination and self-value and what you want to do” (George, 69) but they also liked the inclusion of evidence-based information on how to achieve the lifestyle change: “Most people know it has something to do with their family, but if you have sensible way how to prevent it then that’s what you have to do” (Clare, 65).

Also, professionals valued the work undertaken on HEAL ABC project: “I think this is brilliant, I am very pleased this work has been done in our specific tumor group. There is a huge need for it” (Dietitian 5).

Feedback from the focus groups and interviews are summarized in Table 2 and further comments and suggested improvements are in supplementary material (Table S1). Several common themes identified are detailed in further text.

### 3.2. Identified Themes Based on Focus Group Discussions and Telephone Interviews

#### 3.2.1. Understanding the Behavior Change Concept

Some participants recognized the importance of the lifestyle change: “I think the whole thing is a brilliant idea how to reduce cancer and it is the way to go…” (Lucy, 67). They also perceived that making a change is not an easy task to do: “And it is very challenging at our age to change but I agree it is not impossible” (Lucy, 67).

Interestingly, the concept of behavior change and planning is a very new approach for many people. This concept can be quite difficult for some people to understand: “Well, you say why I want to make this change? Well, I want to save my life, so it is little bit, I do not know, it is an obvious question, so I do not know if it helps me to just write it down” (John, 66). The participants expected the resource to provide specific diet and activity advice “It should be tailored to every person that it is given to, based on the operation that they had” (Paul, 71) and they did not automatically relate to the concept of the booklets providing motivation and a guide to behavioral change.

#### 3.2.2. Contradictions in Fiber and Wholegrains Consumption

Discussions from the focus groups also revealed that some confusion exists around fiber and wholegrain consumption after bowel cancer surgery with or without active treatment. Participants emphasized that they all have unique needs and many of them are extremely careful about fiber and wholegrain consumption. Based on focus groups and interviews, it appears that some people do not differentiate between diet recommendations provided to them immediately after the surgery and what they are able to eat later. This precaution makes people scared to try new foods they might potentially tolerate. “It is difficult to consume lots of fiber if part of the bowel is removed. It was recommended to me after bowel cancer operation not to eat too much fiber as it could not be digested easily” (Jason, 66).

There are also some clear contradictions on fiber for people with colostomy and ileostomy: “On this one, it says that with colostomy there are no restriction on fiber, but dietitians say that there are restrictions. So, it is contradicting what the dietitians say” (Mark, 69). However, dietitians provided a completely different perspective: “We do not restrict even people with ileostomy too much and try to encourage them to have fiber in their diet” (Dietitian 1).

#### 3.2.3. Malnutrition after CRC

For those participants who were older and had problems with maintenance of body weight, the WCRF/AICR guidelines were contradicting dietitians advice on prevention of malnutrition and weight loss in older people: “Sometimes with our weight, you have to eat some of those biscuits and creams” (Sofia, 74).

#### 3.2.4. Overwhelmed by Information

Most participants felt overwhelmed by receiving 11 workbooks in one package, and healthcare professionals stressed that it could be a potential problem for people. “You literally go home with a library of booklets. You are in an information overload and you cannot start processing information until six weeks post-surgery” (Anna, 49). When participants understood that the workbooks would be ‘drip fed’ in reality and that they only received all the workbooks together for the purpose of review, their responses changed. “I think it shouts out right and its good there are individual booklets” (Emma, 44). However, some participants still would like to see more concise version: “I would cut out the repetition and combine booklets together” (Julia, 71). Similarly, professionals suggested to make the resource more compact “Just speaking out loud, could you have just one workbook where patient will set goals and plan their action with pages for all the themes” (Occupational therapist).

#### 3.2.5. Resource Content

Participants appreciated the educational value of the booklets and consideration given to different types of hints and tips given for eating after bowel cancer. For instance, participants found the portion size guides, healthy tips, swaps, and the goal setting combined with the action plan useful. “This is a useful chart for what you need to buy and what you need to store at home”. “I like a Plan Review as a tick chart to know what you have achieved in the week” (Jason, 66). They liked supplementary booklet on fiber and lists of foods they could choose from. “It was good suggestion to try new things. I found some fruit and veg I have never eaten before” (Jane, 41).

Both professionals and people after bowel cancer agreed that the introduction booklet was complicated and suggested that it needed to be simplified. Some participants asked for more evidence and research behind recommendations in addition to what was provided in the booklets. Similarly, professionals agreed that based on an educational level and the “teachable moment”, people after bowel cancer might want to seek more evidence. Also, participants proposed to keep a balance between bad news and restrictions in booklets that target red meat, processed food, sugary foods, drinks, and alcohol consumption. They viewed some information as being too restrictive, which could potentially create resistance towards making a change.

#### 3.2.6. WCRF/AICR Guidelines on Prevention

Some CRC survivors had negative comments about the WCR statements on cancer prevention. They believed that they followed a healthy diet and did enough exercise, but still were diagnosed with cancer. “So, on the front page you say, eat fiber every day to help prevent cancer. Is that right? I think I followed a reasonable diet and I got cancer anyway” (Paul, 71). Hence, they did not like statement such as “eating fiber can help prevent or reduce cancer risk” and did not believe that making dietary changes can help them reduce the risk. The professionals made similar points: “Some booklets, give impression that if you follow this, you will never get cancer, but we know that people still can get cancer because of other factors. I think you should be more careful with the first pages of the booklets. People may think, they caused their cancer” (Dietitian 3). Also, some participants had a very strong belief about physical activity and exercise: “It is like all these activity booklets you can frighten some people. I think there are many reasons why we cannot do exercise” (David, 70).

Most of the CRC survivors had never heard of WCRF organization and did not understand the evidence base of prevention recommendations provided: “What is this World Cancer Research Fund you are referring to everywhere? I have never heard of it” (Emma, 44). Also, the confusing information that survivors receive from TV, magazines, and social media were concerning. “I just wanted to say that along my journey I used to say and what about my diet, oh just a varied diet, if you want to have bacon have bacon. And then all these things come out on the news about what you should and should not been eating, but no one did formally, so I was quite pleased to see something like this” and pointed at the booklets (Eva, 52).

#### 3.2.7. Final Thoughts

Overall, CRC survivors found the HEAL ABC booklets very useful and wanted to use them in their everyday life. They identified which booklets were the most relevant for them and stated that reviewing the booklets prompted them to think about their lifestyle and possibilities for improvements in diet and physical activity. Professionals acknowledged the importance of the evidence-based resource, involvement of CRC survivors and professionals in the resource development and the potential for use in clinical practice.

## 4. Discussion

The importance of lifestyle factors on prevention of cancer are clearly justified and supported with robust evidence. However, without implementation of active behavioral change strategies for the formation of new habits, knowledge of healthy diet and active lifestyle is often insufficient to be translated into behavioral action with positive physiological effects [20].

The feedback on HEAL ABC resource was very positive, insightful, and helped us to understand the needs of people after bowel cancer, but also see the perspective of professionals involved in CRC care or related research. We found that many participants were overwhelmed by amount of information and lack of guidance on improvement of their lifestyle, but also were confused from contradicting information presented by media outlets.

Although CRC survivors identified the resource as useful to improve their lifestyle, many did not understand the concept of behavior change and evidence-based process on how to change behavior. This is in line with a qualitative study showing that when patients are asked about the resource that would help them along the way, most patients cannot identify anything that could help them achieve their goals [17]. Thus, the HEAL ABC resource can provide survivors with a new insight into the action they can take and offer a step by step guide on achieving their goals.

Previous research also identified that cancer survivors often have a very broad idea about their goals, which can be viewed more as outcome goals for their desires rather than specific actions they must take to achieve desired outcomes [17]. This suggests that cancer survivors need help with developing goals that lead to new behaviors and desired results. The participants in our research liked the HEAL ABC resource because it allowed them to set, plan, track, and review their goals, but also guided them on how to achieve their goals by providing ideas on portion sizes, healthy tips, and swaps or lists of foods.

The difficulty to understand the concept of habit formation in the booklets and uniformity among many participants seeking specific dietary recommendations led to discussion on the provision of dietetic advice after surgery. In the UK, not all people after bowel cancer surgery are seen by a dietitian. Often, generic dietary advice is given by other healthcare professionals such as a specialist nurse. A resource that supports diet and lifestyle changes after bowel cancer within the healthcare system might help to support both professionals and cancer survivors. The provision of information will also assist in standardizing advice given while providing a reference source that is evidence-based from the current recommendations as stated by the WCRF/AICR. However, HEAL ABC might be the most effectively used when survivors know their specific dietary needs. The participants were specifically concerned about fiber and wholegrain consumption, and hence with tailored advice by a dietitian prior to using the booklets could strengthen survivor’s confidence in setting goals and improving their fiber and wholegrain intake.

Our research shows that survivors understand the importance of healthy lifestyle and they are willing to change their habits, but when it comes to selection of a specific action, many believe that diet or physical activity does not play a major role in cancer development. A study exploring needs and preferences for dietary support in CRC survivors showed that from 1198 participants only 17.5% reported a need for dietary support, and interestingly this was associated with being younger, living without a partner, having a stoma, diabetes or being overweight or obese [27]. While a recent survey of 208 responders, showed that 15–17% sought dietary advice, 43% looked for information on body fatness and 38% on physical activity [28].

Many survivors were not aware of the WCRF/AICR organization who formulated cancer prevention guidelines. Survivors were informed about heathy recommendations, but not necessarily about recommendations specifically after cancer, and they also justified their own barriers as to why they were not able to follow them. Similar findings were reported in the study on awareness of healthy recommendations in survivors of CRC. The study showed that survivors were familiar with healthy recommendations and identified the effort required for change, as the most common barrier highlighting the need for increased awareness of recommendations relevant to survivors [12].

Lastly, the opinion on structure and lay out of the HEAL ABC resource seems to be underpinned mainly by age and educational level. There are clear differences in desired font size, graphical presentation, and booklet size between younger and older adults. These age differences are not surprising as they were previously reported in relation to decision making processes [30] and lifestyle behaviors [31]. The use of smartphone application with an option to change visual settings might help in addressing preferences of survivors of different age groups. Albeit, there are barriers for older people in access and willingness to use technology which should be considered [32].

## 5. Conclusions

There is a demand for a cost-effective, evidence-based tool which could help people living after bowel cancer in the transition of their motivation into action after cancer. Developed HEAL ABC booklets are a promising tool that can meet these demands. By following a rigorous process in development of the HEAL ABC resource, we are building an evidence-based tool aligned with needs and suggestions of the potential users.

## Figures and Tables

**Table 1:** Characteristics of survivors of CRC.

**General Characteristics *n* = 19**	
**Age**	62 (SD 11.5)
**Gender**	
Female	11 (58%)
Male	8 (42%)
**Ethnicity**	White British
*n* = 13 *	
**Marital status**	
Married	8 (61.5%)
Single	3 (23.1%)
Divorced	2 (15.4%)
**Education level**	
Degree	5 (38.5%)
Higher education diploma	4 (30.8%)
A level/GCSE	3 (23.1%)
No formal education	1 (7.6%)
**Location of surgery**	
Colon	7 (53.8%)
Colon and rectum	6 (46.2%)
**Stoma**	
Yes	4 (30.8%)
No	9 (69.2%)
**Smoking status**	
Active	1 (7.7%)
Past	2 (15.4%)
Never	10 (76.9%)

* Participants who provided information on marital status, education, surgery, and lifestyle. CRC: Bowel or colorectal cancer.

**Table 2:** Positive feedback from participants on HEAL ABC resources.

Theme	People after Bowel Cancer	Professionals
**Structure**		
Organization and explanation of tasks	“Structure clear and helpful” “Similar format in each booklet and familiarity when you open a new book” (Eva, 52).	“The structure is really nice, you explain the WCRF recommendation and then give them the guide and how they can plan their goals” (Dietitian 2).
**Lay out**		
Font	“I really like the font and it’s big enough to read” (George 69).	Not reported
Layout	“I thought it is very attractive, colorful, easy to read, not too much text and appropriate language. It is a good product” (Rose, 65).	“I really like you have the rainbow there to write down the names of fruit and vegetables” (Occupational therapist).
**Content**		
Background information	“It’s good for educational purposes as people have to change their lifestyle you know after operation” (Paul, 71).	“I like it, it is reasonable language and fairly straightforward” (Dietitian 4).
Portions guide	“I found very relevant and useful the portions in red meat booklet, as vegetarian it was useful for me to find out about protein” (Jane,41).	“I like the portion sizes. Everyone knows they should eat 5 a day but they do not know how much to eat” (Dietitian 3).
Healthy tips and swaps	“Useful to get you think about what you can swap” (Jason, 66).	“They have plenty of choices there and different ways to eat” (Researcher 1).
Setting goals and planning	“I like it. You can put in what you want to do and back track your goal with your diary” (Emma, 44).	“I love the way to plan the goals, very smart and easy. Patient can discover by reading the booklet, what they want to do and how” (Research nurse).
Body assessment	“I thought it is very important to have body assessment as I gained quite a lot of weight during my chemo” (Jane, 41).	Not reported
Daily activities and exercise	“Really like exercise booklet, would like to try the exercise and meal alternatives particularly. I would use booklet to improve my lifestyle” (Julia, 71).	“I think your pictures are excellent and the descriptions of the exercises explain the exercises well. I like the exercise record at the back and tips on how to sneak exercises into your routine” (Physiotherapist).
Using the booklets	“I am already using your booklets. I almost wish I could start in hospital and do changes in my diet” (Sofia, 74).	Not reported

## Supplementary Materials

The following are available online at https://www.mdpi.com/2072-6643/11/10/2482/s1, Table S1: Improvements suggestions based on participants feedback.

## Appendix A

Questionnaire for people after bowel cancer.

## Appendix B

Questionnaire for professionals.

## Appendix C

Topic guide for group discussions and telephone interviews.
