# Supplementary material for: Exploring Views of Healthcare Professionals, Researchers, and People Living with and beyond Colorectal Cancer on a Healthy-Eating and Active Lifestyle Resource
Source: Nutrients. 2019 Oct 16;11(10):2482. doi: 10.3390/nu11102482 (PMC6835229; doi:10.3390/nu11102482)
Supplement: Supplementary file 1 [file nutrients-11-02482-s001.zip › Appendix A.pdf]

HEAL ABC

Healthy Eating and Active Lifestyle After Bowel Cancer

We would now like you to complete these questions that tell us about your background.

These details will be kept confidential. If you do not wish to answer some of these questions you do not have to.

Background Information

Please answer each question or tick the relevant box.

1. **What is your age?** *(Please add your age in years)*

   ☐ Years

2. **What is your gender?** *(Please tick one of the boxes)*

   Female ☐ Male ☐ Transgender ☐ Prefer not to say ☐

3. **What is your marital status?** *(Please tick one of the boxes)*

   Single ☐ Living with partner ☐ Married ☐
   Separated/divorced ☐ Widowed ☐

4. **What is your occupation (previous occupation if retired)?**
   (Please write on the line)

----

5. **What is the highest level of education you achieved?**
(Please tick one of the boxes)

- No formal qualifications ☐
- Trade qualification or NVQ ☐
- GCSE level/or equivalent ☐
- A Level/or equivalent ☐
- Higher Education Diploma ☐
- Degree/or equivalent ☐
- Higher degree (MSc/PhD) ☐

6. **What is your ethnic origin?** *(Please tick one of the boxes)*

British ☐
Irish ☐
Other ☐
<u>Mixed</u>
White & Black Caribbean ☐
White & Black African ☐
White & Asian ☐
Other ☐
<u>Chinese</u>
Chinese ☐

<u>Black or black British</u>
Caribbean ☐
African ☐
Other ☐
<u>Asian or Asian British</u>
Indian ☐
Pakistani ☐
Bangladeshi ☐
Other ☐
<u>Other Ethnic Group</u>
Other ☐

7. **What is the total income coming into your household each month?**
(Please tick one of the boxes)

Under £250 ☐ £251 to 500 ☐ £501 to 1000 ☐

£1001 to 2000 ☐ over £2000 ☐ Do not want to answer this question ☐

8. **What are the first 3 digits of your post code?**
(Please write in the boxes)

☐ ☐ ☐

9. **When did you have your colorectal surgery?**
(Please write in the boxes)

Month ☐ Year ☐

10. **Where about in your gut was your surgery?**
(Please write in the boxes)

Bowel ☐
Rectum ☐
Bowel and rectum ☐
Other ☐

11. **Please list all other medical problem you have where you are under a Hospital doctor.** *(Please write on the lines)*

----

12. Do you have a stoma? *(Please tick one of the boxes)*

Yes ☐ No ☐

13. How many of these recommendations do you meet? *(Please tick all the boxes that you would say yes to)*

Would you consider you are a healthy weight for your height? ☐

Are you moderately active for at least 30 minutes a day? ☐

Do you eat 5 potions of fruit or vegetables a day? ☐

Do you have a lot of sweet foods (cakes, biscuits, sugar, sweets) in your diet? ☐

Do you have a lot of foods high in fat in your diet (chips, pies, cakes, cream)? . ☐

Do you have red meat more than three times a week? ☐

Do you drink alcohol? ☐

When you drink alcohol, do you drink more than once a week? ☐

When you drink alcohol do you have more than 2 alcoholic drinks? ☐

14. What is your smoking status? *(Please tick one of the boxes)*

Current smoker ☐
Ex-smoker ☐
Never smoked ☐

Thank you very much for competition of this questionnaire.
