# Supplementary material for: Exploring Views of Healthcare Professionals, Researchers, and People Living with and beyond Colorectal Cancer on a Healthy-Eating and Active Lifestyle Resource
Source: Nutrients. 2019 Oct 16;11(10):2482. doi: 10.3390/nu11102482 (PMC6835229; doi:10.3390/nu11102482)
Supplement: Supplementary file 1 [file nutrients-11-02482-s001.zip › Appendix B.pdf]

HEAL ABC

Healthy Eating and Active Lifestyle After Bowel Cancer

We would now like you to complete these questions that tell us about your background.

These details will be kept confidential. If you do not wish to answer some of these questions you do not have to.

Background Information

Please answer each question or tick the relevant box.

1. **What is your age?** *(Please add your age in years)*

   ☐ Years

2. **What is your gender?** *(Please tick one of the boxes)*

   Female ☐ Male ☐ Transgender ☐ Prefer not to say ☐

3. **What is your occupation?**
   (Please write on the line)

----

4. **What is the area of your expertise in healthcare?**
   (Please write on the line)

----

5. **How many years have you been working in healthcare services?**
(Please write on the line)

6**. What is your ethnic origin?** *(Please tick one of the boxes)*

British ☐
Irish ☐
Other ☐
Mixed
White & Black Caribbean ☐
White & Black African ☐
White & Asian ☐
Other
Chinese
Chinese ☐

Black or black British
Caribbean ☐
African ☐
Other ☐
Asian or Asian British
Indian ☐
Pakistani ☐
Bangladeshi ☐
Other ☐
Other Ethnic Group
Other ☐

8. **What are the first 3 digits of your post code?**
(Please write in the boxes)

☐ ☐ ☐

Thank you very much for competition of this questionnaire.
