# Supplementary material for: Exploring Views of Healthcare Professionals, Researchers, and People Living with and beyond Colorectal Cancer on a Healthy-Eating and Active Lifestyle Resource
Source: Nutrients. 2019 Oct 16;11(10):2482. doi: 10.3390/nu11102482 (PMC6835229; doi:10.3390/nu11102482)
Supplement: Supplementary file 1 [file nutrients-11-02482-s001.zip › Appendix C.pdf]

Topic guide for HEAL ABC focus group

Lay out of the pack

1. What do you think about the layout of the workbook?
2. Was there anything that you did not like about the individual chapters?
3. Have you got any suggestion for improvement?

Structure of the pack

1. What do you think about the structure of the workbook?
2. Was there anything that you did not like about the structure?
3. Have you got any suggestion for improvement?

Content of the pack

1. Were the recommendations explained sufficiently?
2. Have you found the content useful?
3. Is there something you would like to know and it was not provided in the guide?
4. Have you got any suggestion for improvement?

Following the pack (only for survivors)

1. Would you like to try any suggestions from the workbook?
2. Would you use the workbook to improve your lifestyle?
3. Have you got any suggestion for improvement?
