# Supplementary material for: Exploring Views of Healthcare Professionals, Researchers, and People Living with and beyond Colorectal Cancer on a Healthy-Eating and Active Lifestyle Resource
Source: Nutrients. 2019 Oct 16;11(10):2482. doi: 10.3390/nu11102482 (PMC6835229; doi:10.3390/nu11102482)
Supplement: Supplementary file 1 [file nutrients-11-02482-s001.zip › Table S1 Improvements suggestions based on participants feedback.pdf]

Table S1 Improvements suggestions based on participants feedback.

Theme	People after bowel cancer	Professionals
	**Structure**	
Overwhelmed by number of resources	"It's probably too many booklets, I think it could be condensed slightly" [Jason, 66].	"I agree that you can slim it down as you have the information that is repetitive" [Researcher 1].
Title of fibre and wholegrain booklets	Not reported	"I think, you should be really careful, what title you are giving to each booklet. You need to make it appalling that they want to read it" [Occupational therapist].
	**Lay out**	
A4/A5 format	''Maybe reduce the A5 size for booklets for better handling but I like A4 size so perhaps keep it'' [Eva 52].	"I would advise to do A5 leaflets'' [Research nurse].
Cultures and age inclusive	''Clear instructions'' but need photos and symbols representing different ethnicity'' [Julia, 71].	''Make sure there are pictures of food and people of different cultures'' [Dietitian 1].
Playful layout	"At the time, they can feel ever so slightly like a child work sheets'' [Megan, 35].	"Some of these bits I understand why they are there, but I think it undermines the evidence baseness of the workbook" [Occupational therapist].
	**Content**	
Being unique and have specific requirements	"It should be tailored to every person that it is given to, based on the operation that they had" [Paul, 71].	Not reported
Links to websites and services	"I would suggest links to other websites, videos and recipes" [Anna, 49].	Not reported
Complicated introduction booklet	"I think the message is there but it is too much of it." [John, 66]. "I think its good text is big enough and not text heavy" [Rose,65].	"I do think it's too much text when you open it, so I think if it would be in boxes it would look better" [Researcher 2]. "I don't know what you could remove, it's just necessary text there" [Dietitian 6].
More evidence in booklets	"What is this World Cancer Research Fund you are referring to everywhere? I have never heard of it'' [Emma, 44].	"Could you have a page at the end of each booklet with a title something like digging deeper? Some people who are keen on this especially with higher education they might be interested to read it" [Occupational therapist].
Balance bad news and restrictions	"I would make it big that people do not need to miss out on fast food because now they can choose a healthier option even when they go out for fast food meal" [Jane, 41].	"I was thinking it is lot of bad news, and it basically tells me not to eat it. So I was thinking you could try to make it like- it is ok to have a treat from time to time but remember and have a bit of balance" [Dietitian 6].
Appealing pictures of unhealthy foods	Not reported	"One thing I found about the lot of pictures, you make the pictures very appealing" [Occupational therapist].

Setting goals and planning	''There are too many exclamation marks, it come across a bit condescending'' [Anna, 49].	''I think there is fairly lot of research on using this feedback but what I would remove is the exclamation marks'' [Researcher 2].
Upset about WCRF statement on prevention	''So, on the front page you say, eat fibre every day to help prevent cancer. Is that right? I think I followed a reasonable diet'' [Paul, 71].	''Some booklets, give impression that if you follow this, you will never get cancer, but we know that people still can get cancer because of other factors. I think you should be more careful with first pages of the booklets. People may think, oh I caused my cancer because I am not doing this and they become resistant'' [Dietitian 6].
First page in booklets	‘’Content is explained very well, sometimes with too much details’’ [Anna, 49].	''I noticed in all booklets the first page is full of text, and this all information should be there but I can see it’s better to divided it into two pages'' [Dietitian 2].
Contrast in advice provided	''On this one, it says that with colostomy there are no restriction on fibre but dietitians say that there are restrictions. So it is contradicting what the dietitians say'' [Mark, 69].	''For recommendations to use in booklets on fibre – state to follow the advice of your dietitian based on your personal circumstances'' [Dietitian 10].
Contrast in advice provided	''Sometimes with our weight, you have to eat some of these stuff, pointing on sugar booklet – biscuits, cream'' [Sofia, 74].	Not reported
